# Clinical prognostic characteristics of ocular mucoepidermoid carcinoma: a retrospective study

**DOI:** 10.3389/fonc.2025.1720889

**Published:** 2026-01-13

**Authors:** Jing Li, Runzi Yang, Rui Liu, Nan Wang, Hong Zhang, Jianmin Ma

**Affiliations:** 1 Beijing Institute of Ophthalmology, Beijing Tongren Eye Center, Beijing Tongren Hospital, Capital Medical University, Beijing, China; 2 Pathology Department, Beijing Tongren Hospital, Capital Medical University, Beijing, China

**Keywords:** mucoepidermoid carcinoma, ocular, pathology, prognosis, surgery

## Abstract

**Aim:**

This study aimed to characterize the clinical presentation, pathological features, and prognostic indicators of ocular mucoepidermoid carcinoma (MEC) based on an institutional cohort and a systematic literature review.

**Methods:**

A retrospective analysis was conducted including two distinct datasets: six patients with histopathologically confirmed ocular MEC treated at our institution and twenty-one cases identified through literature review. Institutional cases were evaluated for clinical course, treatment, and recurrence, whereas literature-derived cases were summarized descriptively due to reporting heterogeneity and incomplete follow-up.

**Results:**

Patients in the institutional cohort (n = 6) had a median age of 64 years, with no sex predominance. The lacrimal gland was the most frequent primary site, followed by the eyelid and lacrimal sac. The predominant manifestation was a painless orbital mass, while diplopia and visual loss were less frequent. Intermediate-grade lesions were most common, and orbital tissue invasion was histologically confirmed in two patients. Both patients with invasion experienced tumor recurrence, whereas no recurrence was observed among patients without invasion during follow-u No disease-related deaths occurred within this cohort. The literature-derived group demonstrated substantial variability in grading, invasion patterns, and follow-up duration, reflecting selective reporting toward advanced or recurrent cases.

**Conclusions:**

Ocular MEC demonstrates a high tendency toward local recurrence but generally low disease-specific mortality. Orbital tissue invasion appears to be associated with an elevated risk of recurrence in the institutional cohort, underscoring the need for prolonged surveillance. Given the marked heterogeneity of published cases, survival inference should be limited to consistently followed institutional data.

## Introduction

Mucoepidermoid carcinoma (MEC) is a relatively common malignancy. It mainly occurs in the salivary gland, and a few cases affect the breast, pancreas, lung and other organs (1–3). The occurrence of MEC in the eye is rare, for example, lacrimal gland MEC only accounts for 1-2% of lacrimal gland tumors. The average age of onset is around 50 years old, with a greater proportion of cases occurring in women (4). The diagnosis of MEC mainly relies on pathological examination, including tissue biopsy and cytology. MEC is composed of mucous cells, epidermoid cells and intermediate cells. The degree of tumor differentiation was determined by the proportion of mucous cells to epidermoid cells under microscope. The main treatment options are surgical resection, followed by radiotherapy or chemotherapy. MEC is locally aggressive and has a tendency to relapse and metastasize. Due to the low incidence of ocular MEC, systematic analysis of its prognosis is lacking (5, 6). This article reports 6 cases of ocular MEC admitted to our hospital in the past 5 years, and reviews the literature, mainly discusses the clinical characteristic, pathological features and prognostic factors of ocular MEC.

## Methods

### Patients and ethical approval

In this study, patients with ocular mucoepidermoid carcinoma admitted to our hospital from April 2019 to April 2024 were collected. Inclusion criteria were: (1) mucoepidermoid carcinoma diagnosed by histopathology, (2) cases with complete medical history and (3) no other lacrimal gland or systemic malignancy. Exclusion criteria were: (1) cases with missing prognostic information/missing follow-up and (2) metastatic tumors with a history of other systemic malignancies. According to inclusion and exclusion criteria, we included 6 patients with MEC confirmed by pathological examination.

This study adhered to the tenets of the Declaration of Helsinki. Ethics Committee of Beijing Tongren Hospital, affiliated with the Capital Medical University ruled that ethics committee approval was not required for this study owing to the retrospective design. All subjects were fully informed about the purpose of the study, and informed consent was obtained.

### Data collection

We collected the gender, age, main manifestations, tumor grade, tumor sources, orbital tissue invasion, immunohistochemical manifestations, imaging findings, treatment methods, follow-up and outcome of the patients. Tumor recurrence or metastasis was determined by imaging or pathological examination. In this study, the term "orbital tissue invasion" was used to describe histopathologically confirmed tumor extension beyond the primary epithelial or glandular origin into adjacent ocular and orbital structures. This included direct infiltration of extraocular muscles, orbital fat, eyelid margins, conjunctival tissues, scleral or periscleral planes, and perineural pathways. Cases showing only intra-glandular spread without extension into these structures were classified as non-invasive. Detail can be seen in **Table 1**.

**Table 1:** Demographics, presenting symptoms, histological and immunological features, treatment strategies, and prognosis of 6 patients.

Case	Gender	Age	Tumor sources	Clinical feature	Grade	Surrounding tissue invasion	MAML2	Treatment	Follow-up (months)	Recurrence/metastasis	Death (moutns)
1	Female	60	Eyelid	Mass	High	Eyelid, intraorbital structures	NA^1^	Surgery	4	Recurrence	No
2	Male	64	Lacrimal sac	Mass	Medium	Positive	Negative	Surgery+ radiation therapy	17	No	No
3	Female	63	Lacrimal gland	Mass	Medium	Negative	NA	Surgery	10	No	No
4	Male	83	Lacrimal gland	Mass	Medium	Intraorbital structures	NA	Surgery	41	Recurrence	No
5	Male	55	Lacrimal gland	Swelling	Medium	Negative	NA	Surgery + radioactive seed implantation	47	No	No
6	Female	59	Eyelid	Mass	Medium	Negative	Positive	Surgery	1	No	No

^1^NA stands for not available.

### Literature review

In addition, PubMed, Web of Science, China National Knowledge Infrastructure, the Chinese Science and Technology Periodical Database, and WanFang Data databases were searched by computer to identify the records of patients with ocular MEC. Studies were reviewed from the establishment of the database until publication in April 2024. Search terms include "mucoepidermoid cancer," "ophthalmology," "eyelid," "lacrimal gland," and other related terms. Inclusion exclusion criteria were as above. In addition, we excluded articles published in languages other than English.

## Results

### Baseline characteristics and clinical features

Six patients with histopathologically confirmed ocular mucoepidermoid carcinoma were included in the institutional cohort. The median age at diagnosis was 64 years (range, 55-83), with an equal sex distribution (3 males, 3 females). The lacrimal gland was the most common primary site (3/6, 50.0%), followed by the eyelid (2/6, 33.3%) and lacrimal sac (1/6, 16.7%). The predominant presenting symptom was a painless mass, observed in five patients, whereas one patient reported periocular swelling.

### Pathological features

**Figure 1:**
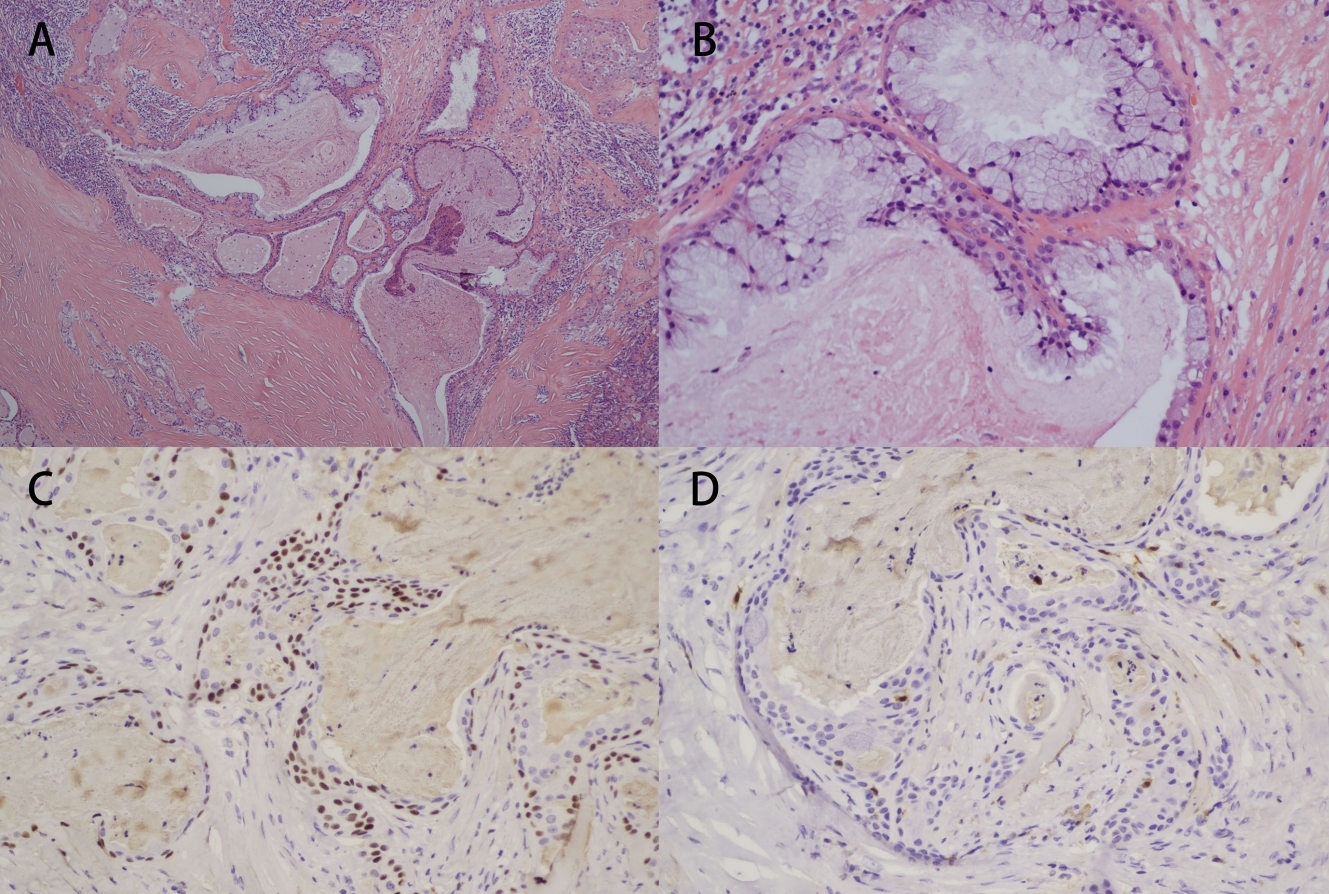
Pathological and immunohistochemical images of Patient 3. **(A)** (Hematoxylin & eosin [HE] staining; 40×) Tumor cells are arranged in solid sheets, nests, ducts, or saccular structures. **(B)** (Hematoxylin & eosin [HE] staining; 200×) The mucous cells are columnar or cup-shaped, with foamy cytoplasm, light staining, basophilic, and small nuclei located at the base. **(C)** Immunohistochemical staining showing positive expression of P63 (200×). **(D)** Immunohistochemical staining showing negative expression of S100 (200×).

**Figure 2:**
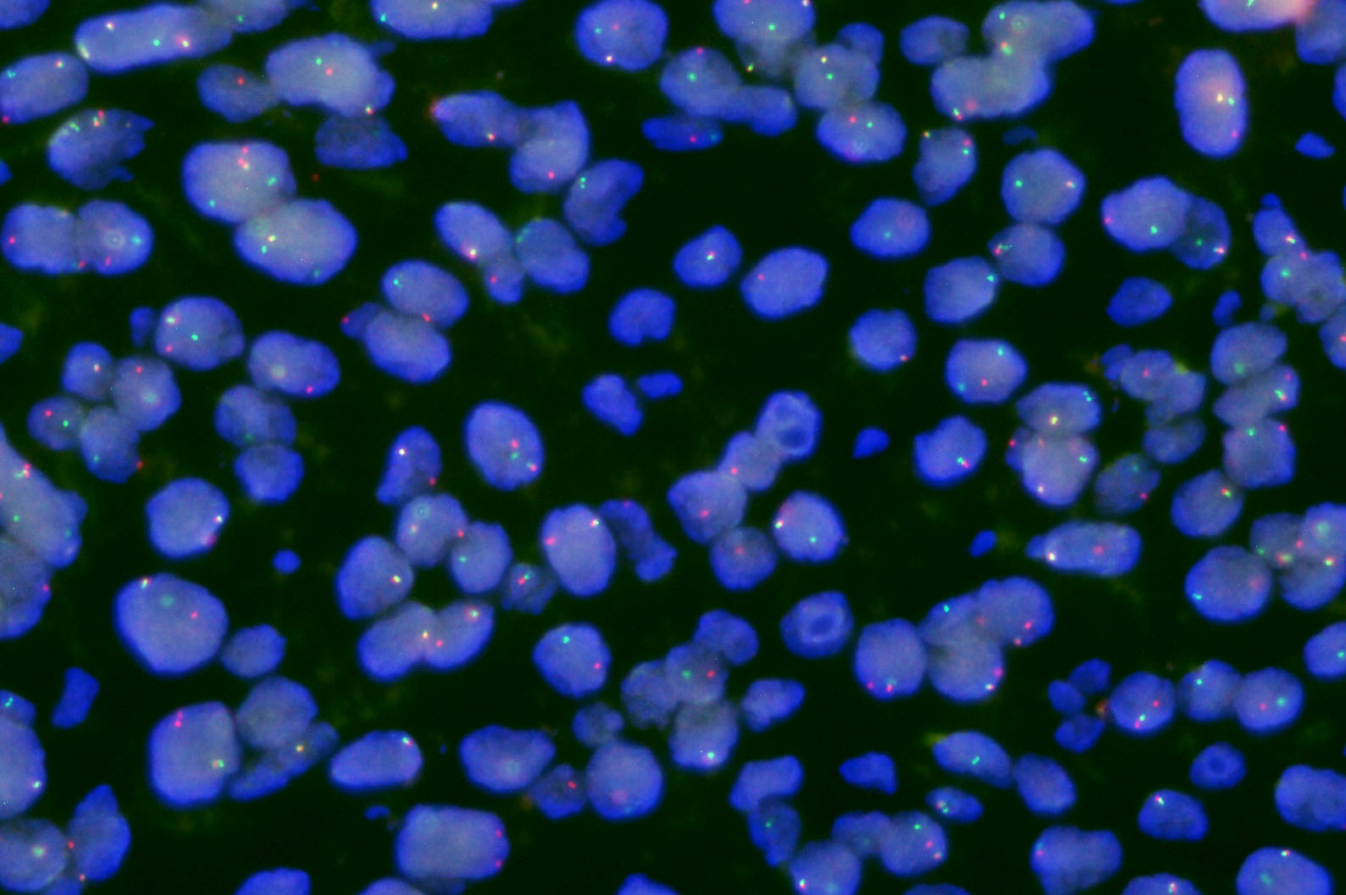
MAML2 gene rearrangement detection (Fluorescence *in situ* Hybridization) images of patient 6. Green fluorescence labeled 3'MAML2 (11q21) probe, red fluorescence labeled 5'MAML2 probe. The normal signal mode is 2F, and the typical positive signal mode is 1G1R1F(G is a green signal, R is a red signal, and F is a yellow or green and red superimposed signal). Results: Cell signal 1G1R1F accounted for 48%, 1R1F accounted for 18%, 1G1F accounted for 8%.

Pathological assessment demonstrated mainly intermediate-grade tumors (4/6, 66.7%), with one high-grade lesion. Orbital tissue invasion was identified in two cases (33.3%), involving the intraorbital structures and eyelid margins, respectively (Detail can be seen in **Figure 1**). MAML2 rearrangement testing was available in two patients, yielding one positive and one negative result, and was not associated with recurrence due to sample size constraints. (Detail can be seen in **Figure 2**). Different cell and tissue structures are different in different tumors and within the same tumor.

### Treatment and prognosis

All patients underwent surgical excision as the primary treatment modality. Three patients received adjuvant therapy, including radiotherapy (n=2) or radioactive seed implantation (n=1). The median follow-up duration was 18 months (range, 1-47). Tumor recurrence occurred in two patients (33.3%), both of whom had documented orbital tissue invasion. No recurrence or metastasis was noted in patients without invasion. No disease-specific deaths were recorded during the observation period.

### Literature review

**Figure 3:**
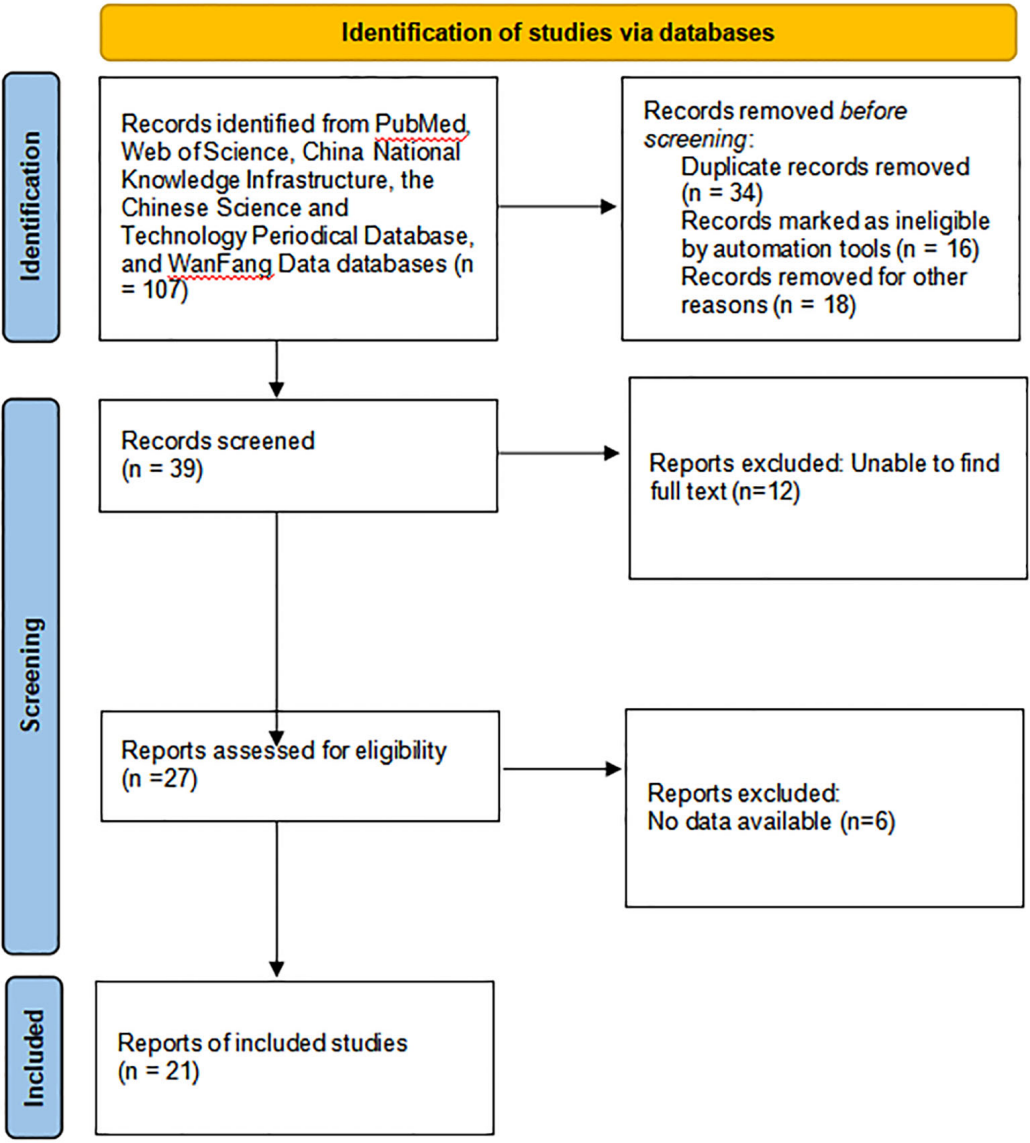
The detailed screening and selection process of 21 cases from the literature review.

We eventually obtained 21 MEC cases that met the criteria by searching the database. The detailed screening and selection process is illustrated in **Figure 3**.

A total of 21 previously reported cases of ocular mucoepidermoid carcinoma were identified through systematic literature review. The majority of cases involved the lacrimal gland (8/21, 38.1%) and conjunctiva (7/21, 33.3%), followed by the eyelid (4/21, 19.0%) and lacrimal sac (2/21, 9.5%). Clinical presentations were predominantly nonspecific, with mass formation as the leading symptom. Less common manifestations included proptosis, epiphora, diplopia, visual loss, and periocular swelling.

Tumor grading varied across reports, with high-grade lesions documented in 6 cases, low-grade in 3 cases, whereas the remaining reports did not provide clear histological grading. Orbital tissue invasion was described in 9 patients (42.9%), most commonly involving perineural structures, extraocular muscles, orbital fat, sclera, and local lymphovascular channels. Molecular profiling was rarely performed, and MAML2 rearrangement status was available in only one case, with a positive result.

Surgical excision was the predominant treatment modality, with adjuvant radiotherapy reported in 9 cases and chemotherapy in one. Enucleation was performed primarily in cases with extensive orbital involvement or destructive invasion. Follow-up duration ranged widely (3–48 months). Recurrence or metastasis was reported in 5 cases, including one case with systemic dissemination.

Given the heterogeneity of clinical reporting, selective publication of recurrent or high-grade cases, and incomplete follow-up data, the literature-derived cohort was assessed descriptively only and was not included in survival or prognostic modeling. (Detail can be seen in **Table 2**).

**Table 2:** Demographics, presenting symptoms, histological and immunological features, treatment strategies, and prognosis of 21 patients form literature review.

Case	Gender	Age	Tumor sources	Clinical feature	Grade	Surrounding tissue invasion	MAML2	Treatment	Follow-up (months)	Recurrence/metastasis	Death (moutns)
7 (10)	Male	30	Lacrimal sac	Mass	Low	Negative	NA	Surgery + radiation therapy	12	No	No
8 (11)	Female	72	Eyelid	Mass	NA	NA	NA	Surgery	48	No	No
9 (12)	Male	15	Lacrimal gland	Mass, diplopia	Low	Negative	NA	Surgery	24	No	No
10 (13)	Female	76	Lacrimal gland	Proptosis	High	Negative	NA	Surgery + radiation therapy	5	No	No
11 (14)	Male	73	Conjunctiva	Mass	NA	Eyelid, orbital structures	NA	Surgery	24	Recurrence	No
12 (15)	Male	52	Lacrimal gland	Mass	High	levator palpebrae superioris muscle, conjunctiva, lateral tarsal ligament	Negative	Surgery + radiation therapy	8	Recurrence, metastasis	Systemic metastasis (20)
13 (16)	Female	89	Conjunctiva	Mass	NA	NA	NA	Enucleation	12	Recurrence	Cardiac disease(24)
14 (17)	Female	45	Conjunctiva	Mass	NA	Perineural invasion	Negative	Surgery + radiation therapy	36	No	No
15 (18)	Female	62	Lacrimal gland	Proptosis	High	Perineural invasion	NA	Surgery	30	Recurrence	No
16 (19)	Male	53	Lacrimal gland	Ptosis, mass	NA	Perineural invasion	NA	Surgery	6	No	No
17 (20)	Female	68	Eyelid	Loss of vision	NA	Orbital tissue, muscle fibers, sclera, choroid, optic nerve	NA	Enucleation + radiation therapy	3	No	No
18 (21)	Female	13	Eyelid	Mass	NA	Negative	Positive	Surgery	15	No	No
19 (22)	Female	85	Eyelid	Swelling	NA	Negative	NA	Surgery	12	No	No
20 (23)	Male	60	Lacrimal gland	Mass, pain, tearing, loss of vision	High	Negative	NA	Enucleation	15	No	No
21 (24)	Male	33	Lacrimal sac	Swelling	High	Negative	NA	Surgery + radiation therapy	6	No	No
22 (25)	Male	74	Conjunctiva	Mass, redness	NA	Negative	NA	Surgery	12	No	No
23 (26)	Male	56	Conjunctiva	Mass	Low	Negative	NA	Surgery + chemotherapy	10	No	No
24 (27)	Male	58	Conjunctiva	Dimness of vision, redness	High	Lymphovascular invasion	NA	Surgery + radiation therapy	13	Metastasis	No
25 (28)	Male	40	Unsure	Swelling	High	Extraocular muscle, orbital floor	NA	Enucleation + radiation therapy	9	No	No
26 (29)	Female	62	Lacrimal sac	Epiphora	NA	Bone	NA	Surgery	12	No	No
27 (30)	Female	54	Conjunctiva	Ptosis	NA	Cornea, sclera, lacrimal gland, nerves, local lymphatics	NA	Enucleation	13	No	No

^1^NA stands for not available.

## Discussion

This retrospective analysis comprised an institutional cohort of six ocular MEC cases and an additional twenty-one cases retrieved from the published literature. There was no gender bias in the incidence of MEC in the study, and all cases were monocular. The main clinical symptoms were nonspecific, including mass, swelling of the lesion area, visual impairment and so on. Lacrimal gland and conjunctiva are the most common sites. Pathological examination showed that the MEC was composed of eosinophilic mucinous cells, intermediate cells and epidermoid cells in different proportions. The surrounding tissue invasion is common in the ocular MEC. The most frequent sites of invasion were peri-nerve, intraorbital structure, extraocular muscle and local lymphatic vessels. Compared with previously reported cases, which often overrepresent aggressive or recurrent presentations due to case-report selection bias, our institutional cohort allows a more balanced view of the prognostic implication of orbital tissue invasion. Notably, we found that recurrence, metastasis, or mortality were significantly higher in patients with orbital tissue invasion than in patients without it. Therefore, we need to pay close attention to the prognosis of these patients and follow up regularly. Although ocular MEC is prone to recurrence and metastasis, it has a high survival rate. In this study, there was only one patient who died from MEC who discontinued treatment after recurrence and multiple metastases. Most of the patients with recurrence can obtain a better quality of life after treatment. Surgical excision and combined radiotherapy are the most common treatment options. Other treatment options include excision, excision combined with radiotherapy, tumor excision combined with chemotherapy. At present, there is no significant difference in prognosis between these treatment options.

**Figure 4:**
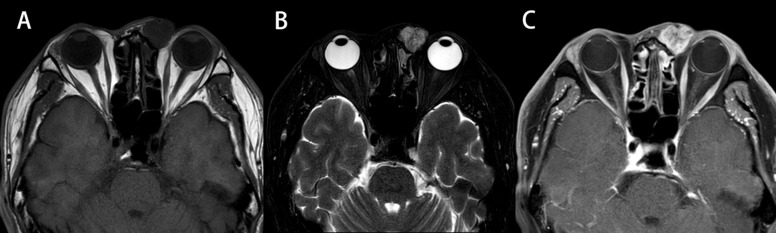
Magnetic resonance imaging of patient 2. Circular masses can be seen in the inner canthus. The intensity of the area in T2-weighted imaging **(B)** is higher than that in T1-weighted imaging **(A)**, and the signals in the area are uneven. After enhancement **(C)**, the enhancement was uneven and obvious. Shallow lobules can be seen at the margin. The lesions involved the lacrimal region and the superior nasolacrimal duct. Bone discontinuity in the right orbital wall. Bilateral frontal sinus, ethmoid sinus and maxillary sinus mucosa thickened.

**Figure 5:**
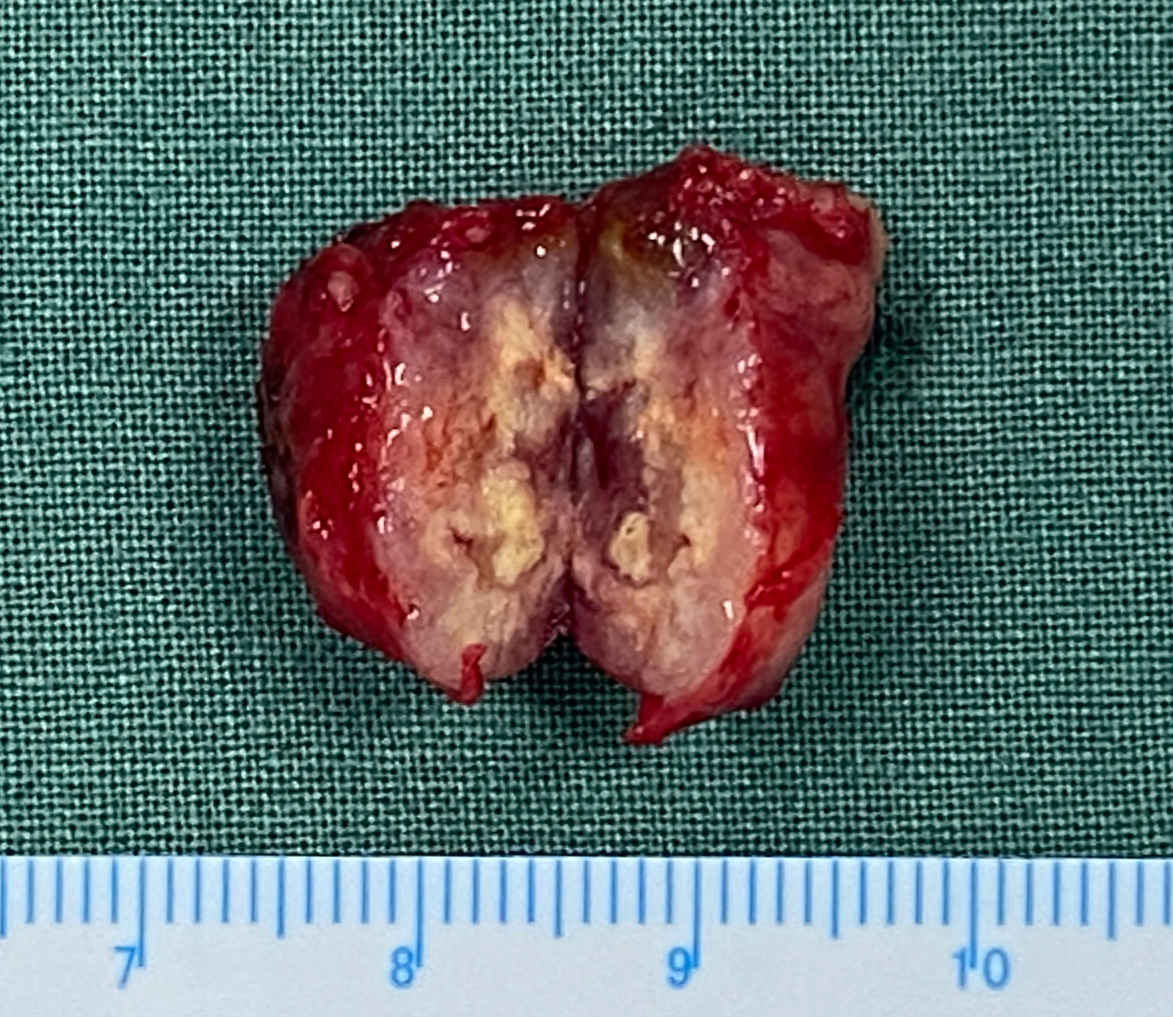
Tumor specimen image of patient 3. The tumor has a capsule with a light pink profile. There are scattered small sacs with pale yellow mucus, blood and translucent areas can be seen.

The diagnosis of MEC depends on pathological examination. It does not show heterogeneity with other ocular malignancies on imaging. (Detail can be seen in **Figure 4**) In terms of tumor appearance, low grade MEC is similar to pleomorphic adenoma except for yellowish mucous visible in the profile. The appearance of high grade MEC tumors is similar to other malignant tumors. (Detail can be seen in **Figure 5**) The pathological features of MEC are epidermoid cells, intermediate cells and mucous cells within the tumor. Although pathology is able to diagnose most MECs, the diversity of MEC morphology presents some challenges. It has been noted that when a component is missing from the MEC, coupled with the presence of distinct cell differentiation, it becomes difficult to detect triphasic cellular populations. At this time, the rearrangement of transcriptional coactivator 2 (MAML2) can be used as the basis for auxiliary diagnosis (7). However, studies have shown that the MAML2 translocation rate of lacrimal apparatus MEC is lower than that of salivary MECs (8). Therefore, MAML2 detection is not widely used in clinical diagnosis of ocular MEC. In this study, patients 2 and 6 in our hospital underwent molecular pathological examination of MAML2 rearrangement, and the results were negative and positive, respectively. Five patients from literature review were tested for MAML2 gene rearrangement, of which 2 were positive (40%) and 3 were negative (60%). Nevertheless, the MAML2 rearrangement of ocular MEC is still of great significance. A study of salivary gland MECs have found that MAML2 rearrangement appears to be a unique molecular feature of Warthin-like MECs, which exhibit mild disease progression and less aggressive bio-behavior (9). This suggests that in addition to diagnostic significance, MAML2 may also be associated with a specific subtype of ocular MEC and better prognosis. Although the results of this study did not show a difference in prognosis, which may be related to the small sample size, this is still a possible direction for future research.

Previous studies have focused on the differences between multiple tumors that occur at specific sites, such as the study of lacrimal gland malignancies. In our study, we investigated specific tumors in the ophthalmic system. In addition, we included the most recent cases and assessed the influence of multiple factors on prognosis. This study has several limitations: First, the disease is rare and the number of clinical cases analyzed is relatively small. Secondly, the follow-up time of cases is not enough to observe the prognosis survival rate. Third, the possible bias caused by racial differences is not considered. We hope to include more cases in the analysis in the future.

In summary, this retrospective study delineates the clinical and pathological spectrum of ocular MEC with emphasis on the prognostic relevance of orbital tissue invasion. In the institutional cohort, recurrence occurred exclusively in patients with documented orbital extension, supporting its role as a potential predictor of adverse outcomes. Despite the tendency toward local relapse, disease-specific mortality remained low. While surgical excision with or without radiotherapy constituted the main treatment approach, no definitive survival advantage between treatment modalities can be inferred due to limited follow-up duration and small event numbers. Close and long-term surveillance is therefore warranted, particularly in cases demonstrating orbital involvement.

## Data Availability

The original contributions presented in the study are included in the article/supplementary material. Further inquiries can be directed to the corresponding author.
